# Development of a rapid test kit for SARS-CoV-2: an example of product design

**DOI:** 10.1007/s42242-020-00075-7

**Published:** 2020-05-11

**Authors:** Zhanfeng Cui, Hong Chang, Hui Wang, Boon Lim, Chia-Chen Hsu, Yejiong Yu, Huidong Jia, Yun Wang, Yida Zeng, Mengmeng Ji, Weizhi Liu, Catriona Inverarity, Wei E. Huang

**Affiliations:** 1 Oxford Suzhou Centre for Advanced Research (OSCAR), University of Oxford, Suzhou, Jiangsu People’s Republic of China; 2 Department of Engineering Science, University of Oxford, Parks Road, Oxford, OX1 3PJ UK; 3 Department of Engineering Science, Institute of Biomedical Engineering, University of Oxford, Oxford, OX3 7DQ UK

**Keywords:** COVID-19, Antigen detection, Rapid test, Product design, SAR-Cov-2, RT-LAMP

## Abstract

We present an example of applying ‘need-driven’ product design principle to the development of a rapid test kit to detect SARS-COV-2 (COVID-19). The tests are intended for use in the field and, longer term, for home use. They detect whether a subject is currently infected with the virus and is infectious. The urgent need for large numbers of tests in field setting imposes constraints such as short test time and lack of access to specialist equipment, laboratories and skilled technicians to perform the test and interpret results. To meet these needs, an antigen test based on RT-LAMP with colorimetric readout was chosen. Direct use of swab sample with no RNA extraction was explored. After extensive experimental study (reported elsewhere), a rapid test kit has been fabricated to satisfy all design criteria.

## Introduction

The COVID-19 pandemic caught almost every country in the world off guard and has made huge impact on every aspect of our society. With over 2 million confirmed cases and 130,000 deaths to date (April 2020), scientific researchers in all disciplines have united to fight this invisible enemy on several fronts. Examples of them include detection and diagnostics, recovery, development of drugs, therapies and a vaccine, rapid production of protective equipment, epidemiology and origin of the disease and its spread and societal impact.

We have developed a rapid test kit for detecting SARS-CoV-2 that can be performed on field without the need for a laboratory or specialist equipment. The technical details have been reported elsewhere [2]. Here, we present the methodology behind the design and development of this test kit as an example of a product design exercise.

Chemical product design methodology has been well developed in recent years [1], which is particularly useful to customer product development. The need-driven product design methodology can be summarised as follows:

- Step 1. Identify the needs.
- Step 2. Gather ideas.
- Step 3. Select ‘the most likely successful idea’ based on the information available.
- Step 4. Test the selected idea. If successful, go to Step 5. If not, go back to Step 3.
- Step 5. Evaluate scalability and manufacturability. If feasible, go to Step 6; if not, go back to Step 3.
- Step 6. Commence manufacture; obtain regulatory approval and access market.

We applied this methodology to guide our development of COVID-19 rapid test kits.

## The need

In late January 2020, some of our team returned to the UK for Chinese New Year holiday. At that time, China was in a state of high alert. In contrast, when passing through the airport in London, we saw no evidence of checks or measures to identify potential carriers. Coming from China at that time, we were shocked by the striking contrast. Now the question came into our minds: ‘what if there was a way to test all passengers waiting to pass through immigration?’. We all remembered the temperature measurement at airports in China after SARS in 2003. A similar rapid test at the airport can help to decide whether the passenger goes to isolation or quarantine (if they test positive) or pass without concerns (if negative). However, there are no biological laboratories or sophisticated instruments and the test would need to be done on-site and in a reasonably short period of time.

Sparked by this idea, a number of useful locations for using on-site rapid tests for COVID-19 were identified, including:

- Primary care clinics or smaller/rural hospitals without laboratory facilities.
- Nursing homes for staff, resident and visitor screening.
- Mass transit hubs (airports and train stations, etc.) for passenger screening.
- Targeted testing of key workers to allow safe return to work.
- Community screening.
- Home testing.

What are common to these application scenarios is the need for rapid tests which do not require access to laboratory, equipment or skilled personnel. The key question is: ‘how can we know an individual is carrying the virus and could be infectious?'. Based on our preliminary study on the information available, we defined the ‘needs’ and set the following specifications:

- Detect SARS-COV-2 (COVID-19) specifically.
- Portable and can be used anywhere.
- Test time 30 min or less.
- Test on-site without transport of samples.
- No need for laboratory and special instruments.
- Easy to operate, with no need for skilled technicians.
- Can test individual and can have sufficient throughput for screening.

## Ideas

Like many real-world problems, there are many possible solutions. What is required is to search for the most suitable and practical solution for the set problem—and in this case, within the shortest time frame racing against the unfolding pandemic. How could we test an individual to confirm whether he/she is infected and infectious? Now all possible ideas and potential solutions are explored.

### Symptom test

COVID-19 is poorly understood. According to the WHO (who.itn, accessed on 15 Apr 2020), ‘The COVID-19 virus affects different people in different ways. COVID-19 is a respiratory disease and most infected people will develop mild to moderate symptoms and recover without requiring special treatment’. Although common symptoms include fever, tiredness and dry cough, it has been proven that many of those infected did not exhibit any symptoms at all and yet were still infectious. Therefore, a symptom-based test, like measuring temperature, cannot be used as a robust diagnostic test for COVID-19.

### Antigen test

The virus, SARS-COV-2, is an enveloped single-strand RNA. The virus exists in the upper respiratory system and hence can be sampled with nasopharyngeal (NP) swabs and oropharyngeal (OP) swabs. RNA detection can be performed using polymerase chain reaction (RT-PCR) or reverse transcription loop-mediated amplification (RT-LAMP) [3, 4]. Both are well-established methods for amplifying DNA. RT-PCR test takes 90–120 min per sample set, while LAMP can be completed with 30 min. The successful detection of COVID-19 virus by either method depends on the design of the primers that can specifically bind the viral RNA and its fragments.

### Antibody test

An antibody test using blood samples (serology-based test) can show whether a person is currently infected or has been in the past. Its limitation lies in its inability to determine whether the person is still infectious. This has important consequences for management of lockdown procedures. Of course, its success depends on identification of the correct antibody.

## Selection of technology

To answer the question ‘how can we test an individual to determine whether she/he is infected and infectious on the spot while she/he waits?’, we first carried out a comparison of antigen, antibody and symptom tests. The decision was simple. We chose to use the antigen test, as only the antigen test could answer this question and meet the identified needs.

Having decided to use antigen test, we searched all available test methods and narrowed them down to PCR and LAMP. The RT-PCR method is the ‘gold standard’ test and widely used worldwide. It involves RNA extraction from the patient swab solution followed by reverse transcription of the RNA and then PCR expansion of the cDNA. The whole process can be automated and high-throughput testing can be performed, which makes it ideal for central test laboratories. However, it is not suitable for field use as it depends on a PCR machine, a laboratory environment for RNA extraction and skilled operators. Additionally, the surge for PCR reagents has created a global shortage of reagents supplies and has limited the test capacities of many test centres. RT-LAMP can detect both RNA and RNA fragments and can deliver the result much faster with easy operation of isothermal amplification of DNA at 65 °C [4]. Thus, LAMP is an obvious choice for its shorter running time as well as the fact that it usually has a lower detection limit. We could take advantage of this and remove the RNA extraction step. If possible, this would greatly simplify the test.

Detailed design and selections had to be made, including:

- What sampling method to use—nasopharyngeal (NP) swabs, oropharyngeal (OP) swabs, saliva?
- What swab solution to use—universal transport solution, buffers, saline?
- How to design the primers so as to uniquely identify the CoV-19 virus?
- How to detect the CoV-19 virus and its mutations?
- How to use reverse transcription (RT) to convert RNA fragments to DNA fragments?
- How to amplify DNA using LAMP?
- How to maintain the LAMP temperature?
- How to display the results?
- How to prevent false positives?
- How to prevent false negatives?
- How to validate the test kits?

It has been emphasised that COVID-19 is poorly understood, even now, and the information related to this virus is very much fragmented. Choices are made based on the most up-to-date information available at each stage and based on best engineering practice.

## Testing, iteration and optimisation

A detailed experimental programme was conducted to test the feasibility of each individual step and process/device integration. Revisions and iterations were made frequently to improve the functionality, usability and practicality. Technical details on the development of the rapid test kit, including clinical validation, can be found in our previous paper [2].

## Manufacturability and practical issues

The rapid test kits must be manufactured following good practice to meet regulatory requirements. Production must also be scalable. Many practical issues have to be considered. One example is that we realised all the bio-reagents could be dried in order to remove the need for cold-chain transport and storage. A proper drying protocol had to be developed, following which testing and optimisation were repeated once more for the whole iteration. Material availability and supply chain are also key considerations.

On top of all this scientific development research, the experiments were repeated multiple times under specific set conditions to test reproducibility, reliability, stability and test shelf life. These additional tests are required to satisfy regulatory requirements, as the product is classified as an IVD (in vitro diagnostic) medical device.

## Summary

**Fig. 1:**
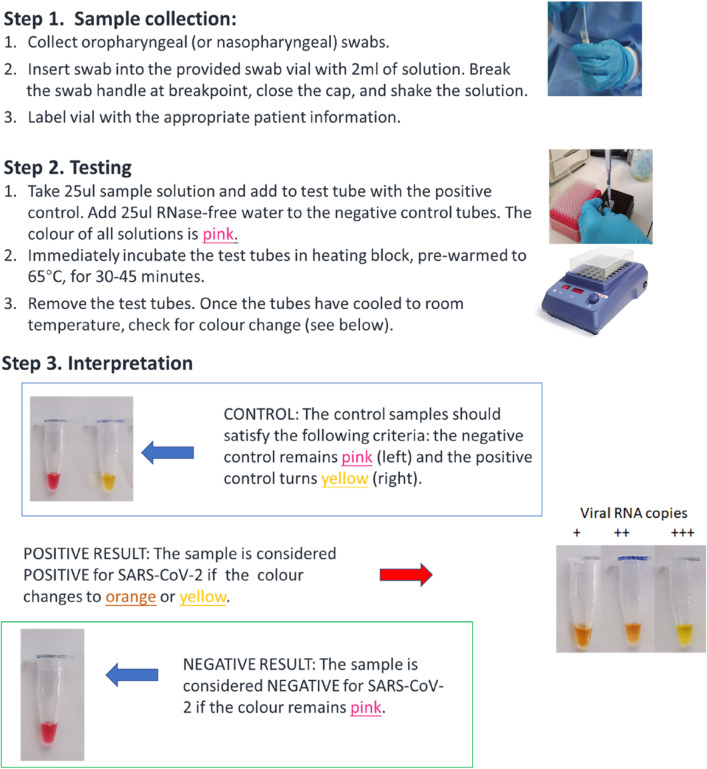
Illustration of the use of the developed rapid test kit for COVID-19

At this stage, we have developed a rapid test kit that fulfil all the identified needs. Its use is illustrated in Fig. 1. Key features are:

1. It uses patient swabs directly.
2. It does not require complicated instruments, with only needing a heating source to maintain at 65 °C. The test even works by adding 1 part cold water to 2 parts boiling water if a heating block is not available.
3. It displays results with colour change, detectable by eye and understandable by lay people.

Following the methodology of product design, the R&D process was highly focused on meeting the defined ‘needs’. In this way, we developed the rapid test kit described here. Naturally, there is plenty of optimisation work, and further tailoring to slightly different applications. The specific ‘needs’ will continue to develop as the virus spreads and affects different regions.
